# Authentication of Algorithm to Detect Metastases in Men with Prostate Cancer Using ICD-9 Codes

**DOI:** 10.1155/2012/970406

**Published:** 2012

**Authors:** Matthew T. Dolan, Sung Kim, Yu-Hsuan Shao, Grace L. Lu-Yao

**Affiliations:** 1 Department of Radiation Oncology, University of Medicine and Dentistry of New Jersey, Robert Wood Johnson Medical School, New Brunswick, NJ 08901, USA; 2 Department of Medical Oncology and Department of Radiation Oncology, The Cancer Institute of New Jersey, New Brunswick, NJ 08901, USA; 3 The Dean and Betty Gallo Prostate Cancer Center, 195 Little Albany Street, New Brunswick, NJ 08901, USA; 4 Department of Medicine, University of Medicine and Dentistry of New Jersey, Robert Wood Johnson Medical School, New Brunswick, NJ 08901, USA

## Abstract

**Background:**

Metastasis is a crucial endpoint for patients with prostate cancer (PCa), but currently lacks a validated claims-based algorithm for detection.

**Objective:**

To develop an algorithm using ICD-9 codes to facilitate accurate reporting of PCa metastases.

**Methods:**

Medical records from 300 men hospitalized at Robert Wood Johnson University Hospital for PCa were reviewed. Using the presence of metastatic PCa on chart review as the gold standard, two algorithms to detect metastases were compared. Algorithm A used ICD-9 codes 198.5 (bone metastases), 197.0 (lung metastases), 197.7 (liver metastases), or 198.3 (brain and spinal cord metastases) to detect metastases, while algorithm B used only 198.5. Sensitivity, specificity, positive predictive value (PPV), and negative predictive value (NPV) for the two algorithms were determined. Kappa statistics were used to measure agreement rates between claim data and chart review.

**Results:**

Algorithm A demonstrated a sensitivity, specificity, PPV, and NPV of 95%, 100%, 100%, and 98.7%, respectively. Corresponding numbers for algorithm B were 90%, 100%, 100%, and 97.5%, respectively. The agreement rate is 96.8% for algorithm A and 93.5% for algorithm B.

**Conclusions:**

Using ICD-9 codes 198.5, 197.0, 197.7, or 198.3 in detecting the presence of PCa metastases offers a high sensitivity, specificity, PPV, and NPV value.

## 1. Introduction

Prostate cancer is a particular diagnostic and therapeutic dilemma because while it is so prevalent among older men, it typically progresses slowly and thus patients often die of other unrelated causes. The five-year relative survival rate for localized and regional prostate cancer is 100%, regardless of race, and 99% for all stages of prostate cancer [1, 2]. Often patients with uncontrolled prostate cancer will have a rising PSA but no clinical symptoms until the development of metastases [3–6]. Prostate cancer metastases most commonly travel to bone, and less commonly to other sites such as brain, bladder, lung, and liver. Once metastases develop, significant morbidity arises and the five-year survival rate falls precipitously to 32% [2]. It is not an exaggeration to say that the development of metastases is a seminal event in the life of a prostate cancer patient and that it often heralds the true onset of morbidity from their disease. The morbidity ensuing from metastases can include severe pain, structural instability of affected bones, spinal cord compression, and neurological compromise [7, 8]. Quality of life is further diminished by the therapeutic measures taken at this point, which may include androgen deprivation therapy, chemotherapy, and palliative radiation or surgery [8–10]. These costly measures often cause considerable urinary, bowel, skeletal, and other physiologic dysfunction, [11–19].

Studies based on Surveillance Epidemiology and End Results (SEER)-Medicare data have become common and have provided important contributions to the prostate cancer literature. They have the advantage of being able to draw on extremely large sets of retrospective patient data. This is especially important in a slowly progressive disease such as prostate cancer, where there is a relative paucity of randomized controlled data on which to draw upon. The endpoint in many SEER studies is death, as that is an easily accessible data point. However, it is clear that development of metastases is an extremely important clinical endpoint as well. To our knowledge, there have been no studies in the literature authenticating an algorithm to detect prostate cancer metastases from SEER-Medicare claims-based data. This study aims to develop and authenticate such an algorithm using ICD-9 diagnosis codes.

## 2. Methods

### 2.1. Study Cohort

This study was conducted on patients who were admitted to Robert Wood Johnson University Hospital (RWJUH), which is the primary teaching hospital of Robert Wood Johnson Medical School and a major referral facility in central New Jersey. The hospital draws from a demographically diverse catchment area with varied patient population. We reviewed the inpatient medical charts of 300 consecutive men (based on initial discharge date from inpatient care) admitted to RWJUH between 1986 and 2007 who had ICD-9 diagnosis code 185 (prostate cancer) for evidence of metastatic disease. For patients with multiple admissions, each available inpatient medical chart was reviewed. Patients without any available medical charts from known inpatient visits were excluded from the study.

### 2.2. Case Identification

We attempted to simulate the SEER-Medicare claims data by accessing the inpatient billing records for RWJUH. In attempting to detect metastases using ICD-9 diagnosis codes, the following codes were used: 198.5 (bone and bone marrow metastases), 197.0 (lung metastases), 197.7 (liver metastases), and 198.3 (brain and spinal cord metastases).

### 2.3. Chart Abstraction and Validation

Paper charts were examined for 300 men who had been inpatients at RWJUH with a history of prostate cancer (as determined by ICD-9 185.0 per RWJUH billing records). Initial inpatient records spanned, 1986 to 2003. All subsequent inpatient charts were examined, so that the complete time frame including all chart reviews was from 1986 to 2007. Electronic medical records (Sunrise Clinical Manager at RWJUH) from 2004 to 2010 were also used for the study. Recorded data on patients included date of prostate cancer diagnosis, initial treatment, presence and site of metastases, date of diagnosis of metastases, mode of verification of metastases, and treatment for metastases. Charts were also reviewed for any coexisting primary neoplasms since these may confound metastatic findings.

Diagnoses of prostate cancer and metastatic disease were confirmed by discharge summaries, admission history and physicals, physician notes, surgical and pathology reports, CT, MRI, and nuclear imaging studies. A single diagnosis of metastatic disease found on medical chart review was considered a positive case. The results of the medical chart review were used as the gold standard against which claims codes were assessed. The chart review was performed by the lead author. An experienced radiation oncologist specializing in prostate cancer reviewed every case in which the chart review did not agree with ICD-9 diagnoses (6 total cases) as well as 20 other randomly selected cases. In every case, the two researchers were in agreement.

All patient data was deidentified, and identifiers were coded in a separate document. This protocol was approved by the RWJUH/CINJ Scientific Review Board and Institutional Review Board.

### 2.4. Statistical Analysis

Based on initial analysis, two algorithms were developed and validated. Algorithm A used ICD-9 codes 198.5 (bone and bone marrow metastases), or 197.0 (lung metastases), or 197.7 (liver metastases), or 198.3 (brain and spinal cord metastases) to detect metastases. Algorithm B used only ICD-9 198.5—since bone and bone marrow are the most common sites for metastases, we wanted to see if this code by itself was sufficient or whether adding codes for the other metastatic sites (Algorithm A) was beneficial. A single ICD code for metastasis was sufficient to be labeled as metastases that is, if using Algorithm A, if there were a patient with ICD 198.5 but not 197.0, that patient was labeled as metastatic. Using the chart review as the gold standard for presence of metastases, sensitivity, specificity, positive predictive value (PPV), and negative predictive value (NPV) for algorithms A and B were determined. The sensitivity was calculated as the proportion of patients who had diagnoses codes for metastases among all patients who had metastases per chart review [20]. The specificity was calculated as the proportion of patients without diagnoses codes for metastases among all patients without metastases per chart review [20]. The PPV was calculated as the proportion of patients with metastases per chart review among all patients who had diagnoses codes for metastases [21]. The NPV was calculated as the proportion of patients without metastases per chart review among all patients without diagnoses codes for metastases [21]. Cohen’s kappa coefficient was calculated to quantify the degree of agreement (overall reliability) on the diagnosis of metastases between the medical records and claims, adjusting for chance agreement [22]. All estimates are presented with 95% confidence intervals (CI). All statistical analyses were performed using SAS statistical software (version 9.2, SAS Institute, Cary, NC, USA).

### 2.5. Algorithm Validation

In order to test whether this algorithm (that was developed using inpatient data) could also be applied to outpatient settings, we applied the algorithm in an independent study cohort and compared the results derived from just inpatient claims versus all claims (combination of hospital, physician, and outpatient claims). The cohort consisted of 29,775 men diagnosed with localized prostate cancer in 1992–2006 identified from the SEER-Medicare linked data. Low risk was defined as Gleason score 2–7 for patients diagnosed in 1992–2002 and Gleason score 2–6 for patients diagnosed in 2003–2006. The rest of the patients were grouped as high risk. These men were aged 66 years or older and did not receive attempted curative treatment within the first year of cancer diagnosis. PADT was defined as patients who received androgen deprivation therapy as primary cancer therapy (no surgery or radiation) within one year of cancer diagnosis. We hypothesized that although the event rate for metastases using just inpatient claims would be lower than that using both inpatient and outpatient claims, the hazard ratios for PADT versus surveillance would be similar regardless of whether just inpatient claims were used versus a combination of inpatient and outpatient claims.

## 3. Results

**Figure 1:**
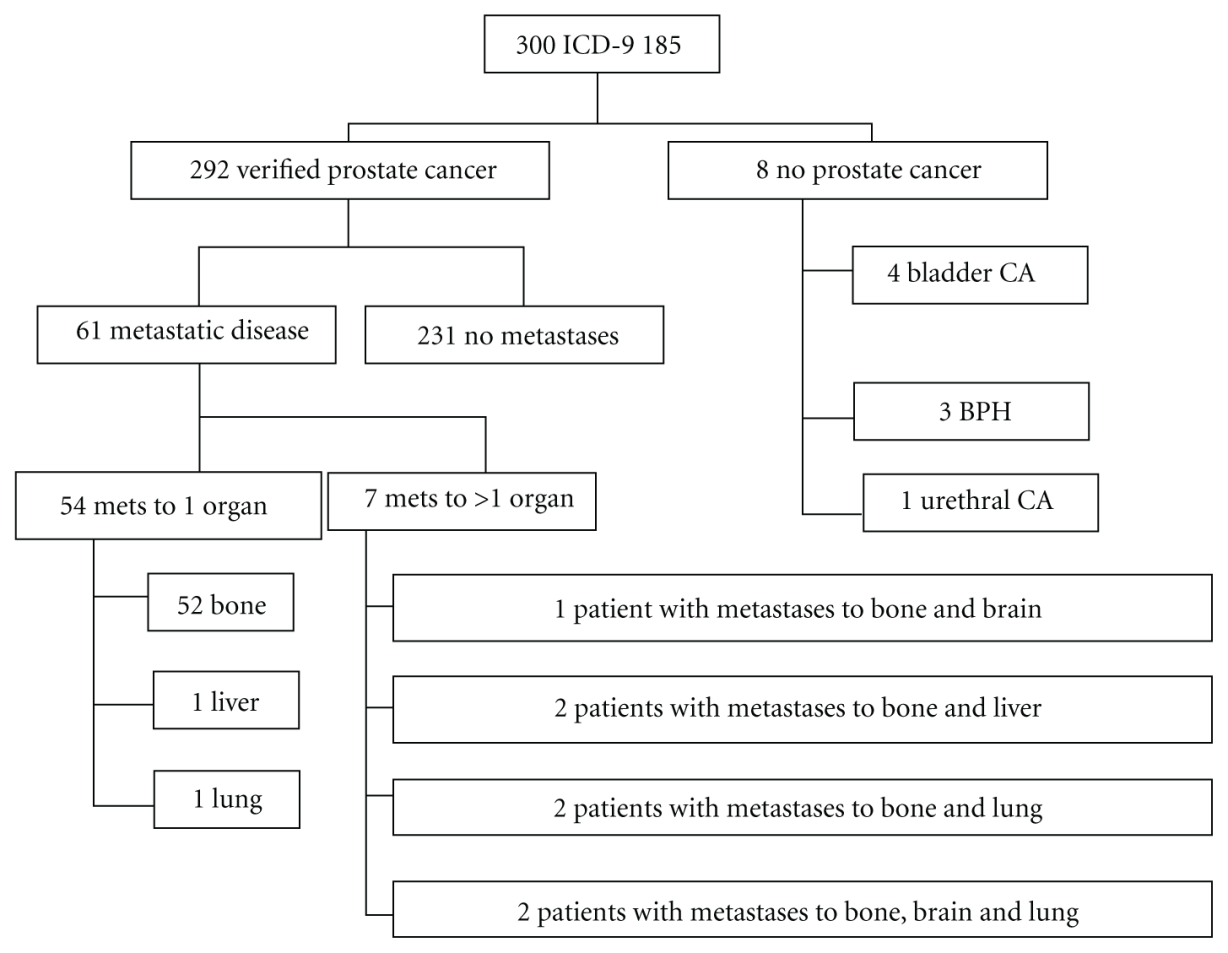
Flowchart of 300 patients who underwent chart review.

The first 300 patients who had ICD-9 185.0, had been inpatients at RWJUH and had inpatient medical charts available, were the subjects of this study. These charts spanned from 1986 to 2003. 1,481 other patients met the first two criteria during this time frame, but were excluded from the study due to unavailability of medical charts. Based on chart review, 8 out of the 300 patients did not have prostate cancer and their data were excluded, leaving 292 patients for analysis (Figure 1). Among the 292 patients eligible for this study, the mean age at initial prostate cancer diagnosis was 66.3 years (range 46–86). The mean length of time between diagnosis and first reviewed RWJUH admission was 1.86 years (range 0–15). 61 patients were found to have metastases on chart review (Figure 1). Metastatic disease was diagnosed a median of 5.25 years after initial cancer diagnosis. Of the 61 patients, 52 patients had only bone metastases, 7 had bone metastases combined with another metastatic site (brain, liver, and lung), and 2 had metastatic sites that did not include bone.

Using algorithm A (Table 1), 58 of the actual 61 patients with verified metastases per chart review would have been detected, for a sensitivity of 0.95 (95% CI, 0.80–0.96). Of 231 patients without metastases per chart review, none were detected by algorithm A, for a specificity of 100% (95% CI, 0.98–1.00). All 58 patients whose diagnoses codes indicated metastases actually had metastases on chart review, for a positive predictive value of 1 (95% CI, 0.94–1.00). Of the 234 patients without diagnoses codes for metastasis, 231 were without metastases on chart review, for a negative predictive value of 0.987 (95% CI, 0.96–0.99). The Kappa statistics (agreement rate) are 96.8% (95% CI, 93–100%).

Using algorithm B (Table 1), 55 of the actual 61 patients with verified metastases per chart review would have been detected, for a sensitivity of 90% (95% CI, 0.80–0.96). Of 231 patients without metastases per chart review, none were detected by algorithm A, for a specificity of 1 (95% CI, 0.98, 1.00). All 55 patients whose diagnoses codes indicated metastases actually had metastases on chart review, for a positive predictive value of 1 (95% CI, 0.94–1.00). Of the 237 patients without diagnoses codes for metastases, 231 were without metastases on chart review, for a negative predictive value of 0.975 (95% CI, 0.95–0.99). The Kappa statistics are 93.5% (95% CI, 88–99%).

As demonstrated above, sometimes metastases on chart review were not picked up by ICD-9 diagnosis coding. Of the three cases where this occurred for algorithm A (Table 2(a)), in one patient, it was equivocal whether he had prostate metastases at all. His prostascint scan showed equivocal, unbiopsied bone metastases, with a differential of metastases versus meningioma. We chose to count him as having metastases though it is unclear if he actually did. In the remaining cases, the patient clearly had metastases and the coding was simply incorrect. For example, one patient had metastases on bone scan and MRI but billing claims were negative for metastases. Similarly, one patient had metastases on bone scan and CT scan but billing claims were again negative. Algorithm B (Table 2(b)) failed to pick up the three patients missed by algorithm A, and in addition, missed three other patients with confirmed metastases on chart review. In two separate cases, radiological imaging showed metastases to only liver or lung—not to bone—and so both these cases were missed by ICD-9 198.5. The last patient had metastases to multiple sites including bone, brain, and lung confirmed by MRI but billing claims were negative.

Table 3 presents the results when metastases of algorithm A (ICD-9 198.5 or 198.3 or 197.0 or 197.7) were applied to a SEER-Medicare cohort and we compared the results when just inpatient claims versus all claims (hospital, physician, and outpatient) were utilized. As we hypothesized, though the event rate for metastases is lower when using just inpatient claims, the resultant hazard ratios for metastases among men treated with primary androgen deprivation versus surveillance were similar regardless of whether only inpatient claims versus all claims were used. The adjusted hazard ratios for low-, high-risk, and all risk patients using all claims were 1.62, 1.65, and 1.66; the corresponding hazard ratios using hospital claims alone were 1.65, 1.55, and 1.64. These results suggest that the developed algorithm is applicable for calculating hazard ratios in the outpatient as well as inpatient settings.

## 4. Discussion

This is the first study that authenticates a SEER-Medicare claim-based algorithm to detect metastases from prostate cancer. It demonstrates that using algorithm A (ICD-9 198.5 or 197.0 or 197.7 or 198.3) offers a very high sensitivity (95%), specificity (100%), PPV (100%), and NPV (98.7%). Algorithm B (ICD-9 198.5) had a slightly worse sensitivity (90%) and NPV (97.5%). As Seer-medicare studies become more important in the prostate cancer literature, we believe that having a validated algorithm for detection of metastases may improve the clinical relevance of some of these studies.

Development of metastases may be the most clinically significant endpoint in prostate cancer prior to death, for several reasons. Most importantly, it heralds an accelerated onset of bone pain, fractures, hypercalcemia, and possible spinal cord compression [18]. Furthermore, metastases occur a median of five years prior to death, so it is for an extended period of time that these patients must deal with metastases, subsequent therapies, and the morbidity associated with both [23]. Finally, development of metastases carries a staggering financial cost. Konski reported that the mean costs for palliative pain medications, single-fraction palliative radiation, multifraction palliative radiation, and chemotherapy were $11,700, $11,900, $13,200, and $15,300, respectively, [24].

In order to know the accuracy of the developed algorithm, we abstracted diagnoses of metastases from SEER-Medicare dataset by utilizing algorithm A and further compared the metastasis rate with a patient cohort reported by Zelefsky et al. [7]. The patient cohort reported by Zelefsky includes 2380 men with prostate cancer and was followup regularly after treatment. The 8-year metastasis-free survival was 92.5% for radical prostatectomy patients and 91.5% for radiation patients in SEER-Medicare dataset, compared to the 97% for radical prostatectomy patients and 93% for radiation patients in this patient cohort, respectively. Given patients in SEER-Medicare are much older than the other cohort, the metastasis rates estimated from our algorithm are fairly comparable with other studies.

This study had certain limitations. Principal among these may be that we looked at an inpatient as opposed to an outpatient population. So presumably the patients in this study had more advanced disease and more prevalent metastases than in the SEER-Medicare database, which has both inpatient and outpatient data. We chose an inpatient population because, logistically, we had access to an inpatient database (inpatient charts, EMR, and inpatient billing records from RWJUH), whereas we did not have the equivalent outpatient records, and also because an outpatient study would necessarily be much larger than this one to detect a similar number of metastases. We certainly would support an outpatient metastases authentication study, but recognizing that it would be a significant undertaking. Recently there was such a study reported utilizing the Danish medical registry [25], which includes both inpatient and outpatient encounters. They also used medical records as the gold standard and used an algorithm consisting of ICD codes for bone metastases and also for skeletal related events. For prostate cancer, they found a sensitivity of 0.54 (95% CI: 0.39–0.69), specificity of 0.96 (95% CI: 0.87–1.00), PPV of 0.93 (95% CI: 0.76–0.99), and NPV of 0.71(95% CI: 0.59–0.81) [25]. Although the sensitivity in the Danish study was much less than in this study, it is difficult to draw any firm conclusions. This database is very different from SEER-Medicare or the RWJUH database, as 46% of these randomly selected prostate patients reportedly developed metastases, which is higher even than in our inpatient cohort. It is also unclear, given that Denmark has free healthcare for all citizens, whether Danish physicians have as much incentive to bill correctly as US physicians, who typically will not be reimbursed without proper documentation. So how useful is algorithm A when applied to the SEER-Medicare database (which includes both inpatient and outpatient data)? Though there likely would be an underestimation of actual metastases, we feel that the true value of this algorithm is not in estimating the absolute number of resultant metastases for a given therapy, but in comparing hazard ratio for metastases given 2 different therapies. For example, applying algorithm A to prostate cancer patients treated with primary androgen deprivation (PADT) in the SEER-Medicare database would likely result in an underestimation of the absolute value of metastases. However, that underestimation of metastases likely applies to other therapies (such as surveillance) as well, so that the hazard ratio of metastases when comparing PADT versus surveillance likely is valid. In fact when we did this exercise for PADT versus surveillance, the resultant hazard ratios were nearly identical regardless of whether inpatient + outpatient or just inpatient claims were used. Though ultimately, only a true outpatient authentication can fully validate an algorithm for metastases in that setting, we feel that this algorithm does have some merit when comparing therapies for metastases.

Another limitation may be that this study involved records of patients admitted to only one hospital (RWJUH). Clearly, different hospitals, care givers, or billing departments may perform differently in terms of accurately coding metastases. The previous argument can be applied again that when comparing metastases resulting from two different therapies, differences in coding accuracy should cancel each other out, leaving a valid hazard ratio. Another potential limitation might be that we used just ICD-9 diagnosis codes, without adding any procedural codes such as those for ADT, palliative radiation, spinal cord decompression, or IV bisphosphonates. Given the high sensitivity, specificity, PPV, and NPV in our study, these procedural codes likely would not have added much in this purely inpatient population. While procedural codes could potentially improve the sensitivity in the outpatient setting, they likely would create more false positives as well, for example, the relatively common scenario where a patient goes on ADT for a rising PSA prior to developing metastases. Other minor issues were that three patients had coexisting primary neoplasms but this likely had minimal effect on the study. Of these three patients, only one was found to have metastases and was detected using both algorithms. Although possibly of nonprostate origin, this single metastatic case likely had a minimal impact on the reported outcomes.

## 5. Conclusions

In this study, using ICD-9 diagnosis codes 198.5, 197.0, 197.7, or 198.3 to detect the presence of prostate cancer metastases offered a high sensitivity, specificity, PPV, and NPV. Though this algorithm is unproven in the outpatient setting, we believe it likely has some merit in determining hazard ratios for metastases when comparing different therapies. As metastasis is such a vital clinical endpoint, we recommend future algorithm authentications in the outpatient setting.

## Figures and Tables

**Table 1:** Validity of diagnoses codes of metastases to prostate cancer.

	Claims	Medical chart review	
		Yes	No
Algorithm A (ICD 198.5 or 198.3 or 197.0 or 197.7)	Yes	58	0
	No	3	231
	Total	61	231
	Sensitivity	58/61 = 0.95 (0.86, 0.99)	
	Specificity	231/231 = 1 (0.98, 1.00)	
	PPV	58/58 = 1 (0.94, 1.00)	
	NPV	231/234 = 0.99 (0.96, 0.99)	

Algorithm B (ICD 198.5)	Yes	55	0
	No	6	231
	Total	61	231
	Sensitivity	55/61 = 0.90 (0.80, 0.96)	
	Specificity	231/231 = 1 (0.98, 1.00)	
	PPV	55/55 = 1 (0.94, 1.00)	
	NPV	231/237 = 0.97 (0.95, 0.99)	

**Table 2:** Cases where metastases were missed by ICD-9 algorithms but confirmed on chart review.

(a) Cases where metastases were missed by algorithm A (ICD-9 198.5 or 198.3 or 197.0 or 197.7) but confirmed on chart review	
Patient	
40	Prostascint scan shows equivocal bone metastases; differential includes metastasis versus meningioma. Not biopsied.
207	Bone scan and MRI show bone metastases.
294	Bone scan and CT scan show bone metastases.

(b) Cases where metastases were missed by algorithm B (ICD-9 198.5) but confirmed on chart review	

Patient	

40	Prostascint scan shows equivocal bone metastases; differential includes metastasis versus meningioma. Not biopsied.
207	Bone scan and MRI show bone metastases.
294	Bone scan and CT scan show bone metastases.
3	MRI shows liver metastases.
50	MRI shows bone, brain, and lung metastases.
164	CT scan shows metastases to lung pleura.

**Table 3:** Validation of metastases algorithm comparison of hazard ratio (HR) of metastasis for primary androgen deprivation (PADT) versus surveillance using all claims versus hospital claims.

Cancer risk	PADT events/person-year	Rate per 100	Surveillance events/person-year	Rate per 100	Unadjusted HR (95% CI)	Adjusted HR (95% CI)
Conventional cox multivariate results Metastasis (use all claims)*						

Low risk	1147/40804	2.8	1627/100346	1.6	1.77 (1.64–1.91)	1.62 (1.49–1.75)
High risk	1163/21546	5.4	457/12888	3.5	1.53 (1.37–1.70)	1.65 (1.47–1.85)
All risk	2310/62351	3.7	2084/113234	1.8	2.05 (1.93–2.17)	1.66 (1.55–1.77)

Metastasis (use hospital claims only)*						

Low risk	309/43083	0.7	428/103651	0.4	1.79 (1.55–2.08)	1.65 (1.41–1.92)
High risk	373/22752	1.6	160/13465	1.2	1.39 (1.16–1.67)	1.55 (1.27–1.88)
All risk	682/65835	1.0	588/117116	0.5	2.13 (1.91–2.38)	1.64 (1.46–1.85)

* ICD-9 198.5 or 198.3 or 197.0 or 197.7.

## Abbreviations

CINJ: Cancer Institute of New Jersey
ICD-9: International Statistical Classification of Diseases and Related Health Problems, Ninth Revision
PCa: Prostate cancer
NPV: Negative predictive value
PPV: Positive predictive value
RWJMS: Robert Wood Johnson Medical School
RWJUH: Robert Wood Johnson University Hospital
SEER: Surveillance Epidemiology and End Results
